# Modified medication use in dysphagia: the effect of thickener on drug bioavailability—a systematic review

**DOI:** 10.1007/s41999-023-00896-6

**Published:** 2024-01-27

**Authors:** Jayne Atkin, Christopher Devaney, Yuki Yoshimatsu, David Smithard

**Affiliations:** 1 General Medicine, Kings College Hospital, Kings College Hospital NHS Foundation Trust, Denmark Hill, London, SE5 9RS UK; 2 Pharmacy, Queen Elizabeth Hospital, Lewisham and Greenwich NHS Trust, Stadium Rd, London, SE18 4QH UK; 3 Elderly Care, Queen Elizabeth Hospital, Lewisham and Greenwich NHS Trust, Stadium Rd, London, SE18 4QH UK; 4 CEAR, University of Greenwich, London, UK

**Keywords:** Dysphagia, Swallowing, Thickener, Modified diets, Thickened-liquids, Bioavailability

## Abstract

**Introduction:**

Dysphagia is associated with long-term conditions including strokes, dementia, Parkinson’s disease and frailty. Dysphagia affects 30–40% of the population aged over 65 years-old. Adults with dysphagia often experience long-term conditions requiring multiple medications (often > 5) to manage these. The thickening of liquids is a common compensatory strategy in dysphagia management. Studies suggest that immersion in thickened liquids affects medicines’ solubility in vitro. Clinicians and pharmacists are unaware of the pharmacokinetic/therapeutic effects of thickened liquids on oral medicines. We conducted a systematic review of existing literature on thickeners’ effects on drug bioavailability.

**Methodology:**

We performed a literature search of MEDLINE & EMBASE. Search terms included: dysphagia/thickened diet (EMBASE only)/ bioavailability or absorption of medicines or pharmacokinetics; excluded: NG feeds/animal studies. Studies included: all genders, countries, > 18 years, community and hospital settings. PRISMA guidance was followed.

**Results:**

Five hundred seventy results were found, and 23 articles identified following the reference list review. Following an abstract and full-text review, 18 were included. Most articles evaluated thickeners on dissolution profiles in-vitro, with a few investigating in-vivo. Most studies were single-centre prospective studies identifying that thickeners generally affect dissolution rates of medications. Few studies assessed bioavailability or used clinical outcomes.

**Conclusion:**

Dysphagia and polypharmacy are common in older adults, but little is known about the effects of altering liquid viscosity on the therapeutic effect of most medications. Further larger-scale studies are required to evaluate the therapeutic impact of thickener, on a bigger range of medications, factoring in other variables such as type of thickener, viscosity of thickener and duration of immersion.

**Supplementary Information:**

The online version contains supplementary material available at 10.1007/s41999-023-00896-6.

## Key summary points

**Aim:**

To understand further the effect of thickener on medications and their pharmacokinetic and therapeutic profiles, using a literature search.

**Findings:**

Despite dysphagia and polypharmacy being common in older adults, little is known about the effects of altering liquid viscosity on the pharmacokinetics and therapeutic effect of most medications.

**Message:**

Further work must be undertaken to support clinicians, pharmacists and patients in understanding the effects of thickener.

**Supplementary Information:**

The online version contains supplementary material available at 10.1007/s41999-023-00896-6.

## Introduction

As adults get older there is an increase in the prevalence of polypharmacy [1]. In England, more than 1 in 10 people over 65 years take at least eight different prescribed medications each week. This increases as people get older—to nearly 1 in 4 in the over 85’s [2].

Dysphagia is a geriatric syndrome associated with many common long-term clinical conditions including stroke, dementia, Parkinson’s disease and frailty. Clave et al. report that oropharyngeal dysphagia may affect 30–40% of the population aged 65 years or more. They also report that dysphagia affects more than 50% of people living in nursing homes [3]. In hospitalised older adults, dysphagia rates can vary between 7.9 and 86% [4]. Thickening fluids is an increasingly used strategy to reduce the risk of aspiration. The increased viscosity of fluids compensates for a swallowing deficit by slowing down the flow of the fluid from the mouth to the oropharynx—allowing time for glottis closure [5]. Steele et al. performed a systematic review on the effect of food texture and liquid consistency modification on swallowing physiology and function, they concluded that there is a clear reduction in the risk of aspiration as liquids progress from thin to very thick (studies involving barium swallow under video fluoroscopy in adults with dysphagia) [6]. Evidence has been found to show people with dysphagia are three times more likely to experience medication administration errors than those without dysphagia located on the same ward [7]; that is if they are diagnosed at all as often patients with dysphagia are undertreated and underdiagnosed in many medical centres [8].

Given the overlapping profiles of those patients prescribed multiple medications and those receiving thickened fluids, understanding the interactions between thickened fluids and medications and how this may affect medication bioavailability and therapeutic effect is essential.

Thickening agents can be broadly classified into 3 groups: starch based, guar gum based and xanthan gum based, often chosen based on their cost, availability, consistency and flavour. There is variability in how thick fluids are that patients receive. In 2012 the international dysphagia diet standardisation initiative was founded, and the characteristics of fluids, thickened fluids and foods were described. The level of viscosity of fluids ranges from 0 (thin) to 4 (extremely thick) [9].

There have been a few studies reporting the effect of thickeners on drugs, but no systematic review has been performed. We have conducted a systematic review to answer the question of whether thickened fluids affect the bioavailability of oral medications. Furthermore, the level of thickness of fluids, the type of thickener used and the duration of emersion in thickened fluid affects the bioavailability of medications. Additionally, we have reviewed evidence as to whether the bioavailability and therapeutic effect of various medications are differentially affected by thickener.

## Methodology

**Fig. 1:**
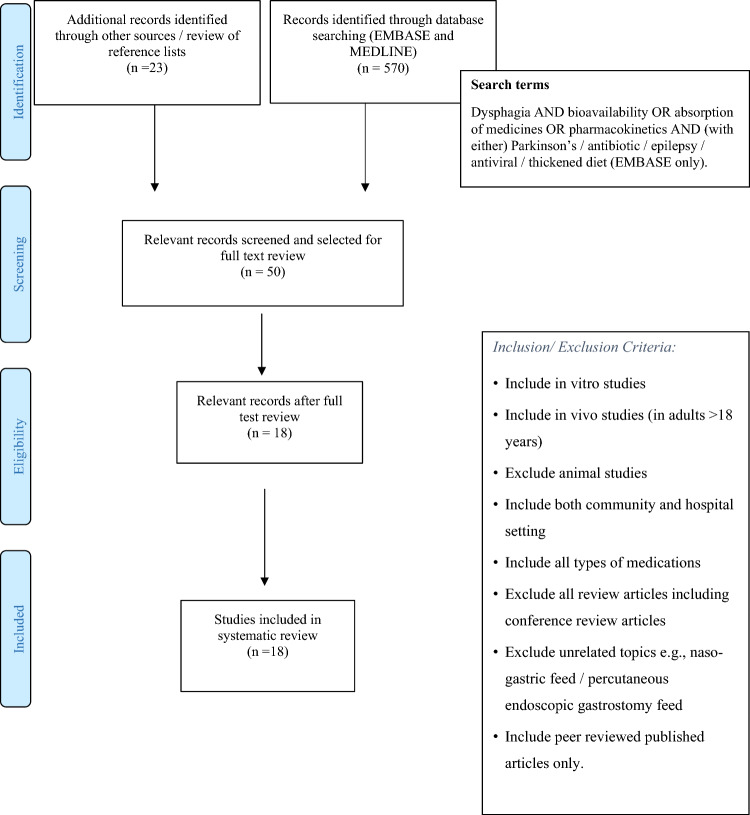
PRISMA flow diagram outlining research methodology

PRISMA methodology was followed to structure the review [10] (see Fig. 1). An experienced librarian performed a literature search of MEDLINE and EMBASE. Search terms included: dysphagia AND bioavailability OR absorption of medicines OR pharmacokinetics AND (with either) Parkinson’s/antibiotic /epilepsy/antiviral/thickened diet (EMBASE only). Two of the authors screened search results (titles and abstracts, duplicates were removed at this stage). No software was used in this process. The terms ‘Parkinson’s’, ‘antibiotic’ ‘epilepsy’ and ‘antiviral’ were included so that we could focus our search on time-critical medications and medications that have a critical impact on clinical outcomes. However, when evaluating search results, we have included all medications due to the small number of studies in this field. We excluded unrelated articles, review articles and conference abstracts. We also excluded animal studies and articles relating to nasogastric/percutaneous endoscopic gastrostomy feeds. All articles included were peer-reviewed published articles. All ages over 18 years were included in both community and hospital settings. No articles were excluded based on the type of medication. Articles describing in vitro experiments were also included. Articles selected for a full review were selected independently and then agreed upon by both authors. Reference lists from these articles were reviewed and further relevant articles were identified. There was no discrepancy in the selected articles. All relevant articles were then summarised according to study design, number of participants, method and main conclusions. The search was undertaken on 26/02/22 with an additional search on 27/09/23. The two investigators scored the papers using the JADAD scoring system. The JADAD scoring system was used as it allowed easy identification of potential bias in the papers we reviewed [11].

## Results

Five hundred seventy results were found in the initial search. A further 23 articles were identified by the authors following reviews of reference lists. Following a review of the abstracts (performed independently by 2 of the authors) 543 publications were rejected. 50 received a full text review and a further 32 were rejected, with the remaining 18 articles included in the review. All studies reviewed were small single-centre studies. The rationale for rejecting studies included study design (e.g., animal studies), study type (e.g., review articles and conference abstracts).

Sixteen papers published between 1984 and 2021 were reviewed (outlined in Table 1). Five studies were in vivo and the remaining eleven studies were in vitro.

**Table 1:** Summary of articles included for review

Author, Year	Title	Study design	Participants	Method	Main conclusions	JADAD score
[12] Manrique et al. 2016	Oral medication delivery in impaired swallowing: thickening liquid medications for safe swallowing alters dissolution characteristics	Single centre prospective study	N/A	Assesses the effect of thickener on the dissolution of paracetamol using Hitachi U-1900 spectrometer Rheology of thickened samples was assessed including viscosity and yield stress	Thickener significantly impacts dissolution of paracetamol The weak gel nature, viscoelasticity and high yield stress impact drug release	0
[13] Takahashi et al. 2020	Effect of xanthan gun-based food thickeners on the dissolution profile of fluoroquinolones oral formulations	Single centre prospective study	3 antibiotics were evaluated	Compared the 15 min dissolution rate with that of non-immersed formulations (control) Floroquinolone film-coated tablets were mixed with starch-based food thickeners, guar gum-based food thickeners of xanthan gum-based food thickeners to observe their appearance	The dissolution profile of levofloxacin film-coated tablets was not affected by xanthan gum-based food thickener (XG- FT) The dissolution of tosufloxacin and ciprofloxacin was delayed after emersion in XG-FT Ciprofloxacin film-coated tablets, after emersion in XG-FT/guar gum FT produced a gel-like precipitate	0
[14] Matsuo et al. 2020	Appropriate usage of food thickening agents to prevent non-disintegration of magnesium oxide tablets	Single centre prospective study	1 medication (magnesium oxide, 5 different brands). 2 thickeners (xanthan gum, guar gum)	Tablets were immersed in food thickener for 1, 10 and 30 min Tablets stored for different durations at the same temp and humidity Disintegration test performed according to the Japanese Pharmacopia (17 edition)	Duration immersed in thickener significantly affects disintegration time Moisture absorbed by magnesium tablets caused a significant delay in their disintegration in water	0
[15] Tomita et al. 2018	Effect of xanthan gum as a thickener in widely-used food thickeners on the disintegration of rapidly disintegrating tablets	Single centre prospective study	Four different concentrations of xanthine gums aqueous solutions (0.2% w/v, 0.4% w/v, 0.6% w/v. 0.8% w/v)	Magnesium oxide tablets were immersed in four different %w/v xanthan gum aqueous solutions for up to 1, 5 and 10 min Following this, their disintegration rates were tested in purified water (In accordance with the described method in Japanese Pharmacopoeia)	Magnesium Oxide tablets that had been immersed in higher concentrations (0.6 or 0.8% w/v) of thickener took longer to disintegrate in water	0
[16] Tomita et al 2017	Effect of food thickener on the inhibitory effect of mitiglinide tablets on post-prandial elevation of blood glucose levels	Single centre prospective study. Cross over study design	*N* = 5	Xanthan gum as thickening agent. Compared disintegration rates with/without emersion in thickener for 1/10 min Compared effect of mitiglinide tab/mitiglinide tab emersed in xanthan gum thickening agent on blood glucose levels after digestion of 75 g glucose	Significant difference in blood glucose levels from administration to 90 min The greatest difference in blood glucose levels is at 60 min (123 mg/dL for emersed tablets Vs 82 mg/dL for non-emersed tablets. *p* < 0.01)	0
[17] Matsuo et al. 2021	Comparison of the effects on tablet disintegration of solvents used to dissolve food thickeners	Single centre prospective study	4 medications were assessed (magnesium, senna, furosemide and aspirin) in 12 different solvents (including water, tea, juice and coffee)	Xanthan gum-based food thickener was added to each solvent and mixed. Line spread test and the pH of the different solvent thickener mixes were taken Additionally, tablets were immersed in a thickener for one minute and disintegration times were recorded The disintegration time for magnesium tablets immersed in a thickener for 30 min was also recorded	Disintegration times of the tablets immersed in thickener for one minute were similar Magnesium tablets immersed in thickener for 30 min had delayed disintegration times. In coffee the disintegration time was 30 min, in all other solvents it was over 60 min Different solvents did not differentially affect disintegration rates	0
[18] Ruis-Picazo et al. 2020	Effect of thickener on disintegration, dissolution and permeability of common drug products for elderly patients	Single centre prospective study	Six medicines (Aspirin, Atenolol, Candesartan, Ramipril, Acenocumarol, Valsartan)	The six medicines had their disintegration rates tested (both with and without thickener) The dissolution rates of three medications were tested in fluid at various pH levels (both with and without thickener)—those where thickener had the most marked effect on disintegration time (atenolol, candesartan and valsartan) Permeability through rate intestine was also investigated however animal studies are not being reviewed in this article	The disintegration rate increases for all medications with the addition of thickener Thickener did not impact the dissolution rate of atenolol at acidic pH but marked effect on dissolution was observed at pH 4.5 and 6.8 In the case of candesartan thickener0 slowed dissolution rates at all pH levels tested. Similar results were observed for valsartan with the exception of pH 1.2 Note only minor effects of thickener were observed on dissolution rates at all pH levels when valsartan tablets were crushed	0
[19] Matsuo et al. 2020	Effects of thickened drinks on the disintegration of various oral tablets	Single centre prospective study	40 tablets	Tablets were immersed in thickener for 1 min. The disintegration time for non-immersed tablets was used as the control. The disintegration time was defined as the time at which the contents of the tablets were released Tablets were grouped into naked tablets, film-coated tablets, orally disintegrating tablets and enteric and sugar-coated tablets	The disintegration time of all orally disintegrating tablets was longer when immersed in thickener than that of non-immersed tablets (but it was less than 2 min for the majority of tablets) The disintegration time for several other tablets was shorter or unchanged	0
[20] Bravo-Jose et al. 2022	Combining Liquid Oral Drugs with Thickener: Compatibility and Changes in Viscosity	Single centre prospective study	45 medicines	The solubility of 45 medicines in the thickened fluid were assessed The amount of thickener added to drugs to achieve various different thickness consistencies was also evaluated	3 drugs (almagate, ibuprofen and macrogol) were physically incompatible with thickener Viscosity measurements indicated that different amounts of thickener needed to be added to different drugs	0
[21] Sugiura et al. 2020	Effect of disintegrants on prolongation of tablet disintegration Induced by Immersion in Xanthan Gum-containing thickening solution – Contribution of disintegrant interactions with disintegration fluids	Single centre prospective study	n/a	Tested 7 different disintegrants (excipients often found in tablets and most crucial for tablet disintegration) Compared disintegration time of control with tablets immersed in 0.9%w/v XTG for 1 min Also compared various tablet properties (and whether these were affected by thickener)	Disintegration time is significantly prolonged after immersion in the XTG solution. The extent of prolongation differs according to the disintegrants contained in the model tablets	0
[22] Huupponen et al. 1984	Effect of Guar Gum, a Fibre Preparation, on Digoxin and Penicillin Absorption in Man	Double-blind study	*N* = 10	Volunteers received either digoxin or penicillin, medication was taken with either guar gum or a placebo preparation (wheat germ) mixed with 200mls of water Serum and urine concentrations of digoxin were measured by radioimmunoassay Serum penicillin concentrations were measured microbiologically according to Reeves et al. (1978)	Mean peak digoxin concentration was lower after guar gum than after placebo granules (0.05 < *p* < 0.1) but similar amounts of digoxin were recovered by 24 h in urine samples A significantly lower mean peak penicillin concentration (*p* < 0.01) was obtained on guar gum compared to placebo. There was no difference in time to reach peak penicillin concentration. 24-h urine samples were not available for penicillin	4
[23] Tomita et al. 2015	Effect of food thickener on disintegration and dissolution of magnesium oxide tablets	Single centre prospective study	Patients who were admitted to hospital wards during 1–15 October 2014 and were taking magnesium oxide (*n* = 147)	In vitro experiment – rate of dissolution of magnesium oxide tablet was compared in two different thickeners (xanthan gum and guar gum) each at three different concentrations. Tablets were immersed for 30 min, and the dissolution rate was compared to control 21 patients were observed to be using thickening agents. Whether they disintegrated it in a small amount of water prior to thickener was observed (15/21). The dose taken was observed. Whether they took an additional laxative was observed	The dissolution rates for immersed tablets were lower than for non-immersed tablets. Concentration of thickener differentially affects the dissolution rate The mean magnesium dosage was 1705 mg/day in those who took it with a thickened fluid without disintegrating it first, 1122 mg/day in those who disintegrated then took it with thickened fluid, and 1380 mg/day in those who took it with thin fluids. 31 out of 126 (25%) patient who did not use thickeners used an additional laxative, whilst 2 out of 6 (33%) who took magnesium with thickened fluids without disintegration and 9 out of 15 (60%) of those who took magnesium with thickened fluids after disintegration used another laxative in addition to magnesium	0
[24] Tomita et al. 2016	Effects of food thickeners on the inhibitory effect of voglibose oral-disintegrating tablets on post-prandial elevation of blood sugar levels	Single centre prospective cross-over design study	*N* = 9	Participants were fasted for 10 h. Control and study drugs were given 7 days apart. Study drug was voglibose tablet that had been immersed in a 3% thickening agent for 10 min (the control drug was not immersed). Participants were administered 100 g sucrose. Blood glucose levels were checked between 0 and 120 min	There was a significant difference in blood glucose levels (p0.01) between taking the immersed tablet Vs control. The difference was highest at 105 min after taking the sucrose solution. The blood glucose tablets after taking the immersed tablet were 106.2 mg/dL Vs 90.8 mg/dL for control	1
[25] Tomita et al. 2019	Effect of food thickener and jelly water on the pharmacokinetics of levofloxacin orally disintegrated tablets	Single centre prospective study. Cross-over study design	*N* = 4	Disintegration rates after emersion in thickener/jelly water were compared to the control Dissolution rates (after emersion in thickener/jelly water Vs control) in various fluids (at different pH) were compared Compared time taken to reach maximum systemic levofloxacin concentration after emersion in thickener/jelly water (Vs control)	Emersion in thickener significantly effects disintegration and dissolution rates No significant difference in time taken to maximum systemic concentration No significant difference in maximum systemic concentration	0
[26] Watanabe et al. 1992	Factors affecting prednisolone release from hydrogels prepared with water-soluble dietary fibres, xanthan and locust bean gums	Single centre prospective study	n/a	Assessed the release behavior of prednisolone from hydrogels prepared with xanthan and locust bean gums Release behavior was analyzed spectrophotometrically using 5mls of hydrogel to quantify the amount of prednisolone Solubility and viscosity of the hydrogels were also measured	The amount of released prednisolone decreased with increasing gum concentration	0
[27] Manrique et al. 2014	Crushed Tablets: Does the Administration of Food Vehicles and Thickened Fluids to Aid Medication Swallowing Alter Drug Release?	Single centre prospective study		Compared 5 commercial thickening agents at 3 thicknesses (mild level—150, moderate level—400, extremely thick level—900) by assessing their effect on the dissolution of atenolol in 900 mL of pH 1.2 simulated gastric fluid without enzymes (USP dissolution test apparatus II was used) (Also tested other crushed medicines and whole tablets with various different drinks they may be taken with)	At thickness level 150 non-significant delay in the dissolution of atenolol was observed At 400 there was variability in brands in the dissolution At 900 all thickened fluids retarded dissolution when compared to whole or crushed tablets in water (even at 3 h—total duration of the experiment)	0

The in vitro studies mostly evaluated the disintegration and dissolution times of various medicines, comparing control medicines to medicines that had been immersed in thickened fluid for various periods of time. The number of medicines studies ranged from 1 to 45. Endpoints in the in vivo experiments included serum/urine concentrations of medication and therapeutic effect (e.g., serum blood glucose levels when evaluating the effect of thickener on diabetic medications).

### The differential effects of type of thickener in vitro

In 2014 Manrique et al. [12] compared 5 commercial thickening agents at 3 thicknesses (mild level, moderate level and extremely thick level) by assessing their effect on the dissolution of atenolol. At ‘mild’ thickness level no significant delay in the dissolution of atenolol was observed compared to the control. At ‘moderate thickness’ there was variability between brands on dissolution rate and products based on xanthan gum were noted to delay dissolution. Extremely thickened fluids retarded dissolution rates [12]. In 2020 Takahashi compared fluoroquinolone film-coated tablets mixed with starch-based food thickener and xanthan gum-based food thickener—finding that starch-based thickener resulted in a more ‘evenly dispersed’ product [13]. Matsuo’s study in 2020 also bore out the differential between thickeners by comparing guar gum to xanthan gum finding that emersion in guar gum increased disintegration time of magnesium oxide tablets although the study was powered to compare thickeners to the control making the significance unclear. Furthermore, this difference was only noted in some, but not all brands, of magnesium oxide tablets [14].

### The differential effects of thickened fluid viscosity

In 2018 Tomita found that magnesium oxide tablets that had been immersed in higher concentrations (0.6 or 0.8% w/v) of xanthan gum thickener took longer to disintegrate in water compared to tablets immersed in 0.2 and 0.4% w/v [15]. The effect of thickener concentration was also demonstrated in Manrique’s study in 2014 (as noted above) [12].

### The effect of the duration of time emersed in thickened fluid

Tomita’s study in 2017 demonstrated that Glufast tablets immersed in xanthan-gum thickener for 1 min had a shorter disintegration time compared to tablets immersed in thickener for 10 min however the significance of this was unclear as the study was powered to compare both groups to the control (i.e., not immersed in thickener) [16]. In 2018 the same group once again demonstrated this principle, this time with magnesium oxide tablets. They found that tablets immersed in 0.6% thickener for 10 min had a significantly longer dissolution time than those immersed for 5 min. Similar results were born out for tablets immersed in 0.8% thickener [15].

In 2020 Matsuo’s group noted that immersion of magnesium oxide tablets for 10 and 30 min in xanthan and guar-gum-based food thickening agents caused disintegration delay and non-disintegration, however, tablets immersed for 1 min only quickly disintegrated [14]. In 2021 when the group were comparing the effect of different solvents, they found that magnesium tablets immersed in a thickener for 30 min had delayed disintegration times compared to 1 min [1].

### The differential effect of thickening different types of liquids

Ruis-Picazo’s study in 2020 compared the dissolution rates of medications including atenolol, candesartan and valsartan. They investigated whether the pH of the thickened fluid affected the dissolution rate. They found that thickener did not impact the dissolution rate of atenolol at pH1.2 but had a marked effect on dissolution rate observed at pH 4.5 and 6.8 compared to control. In the case of candesartan, thickener slowed dissolution rates at all pH levels tested. Similar results were observed for valsartan with the exception of pH 1.2 [18]. Matsuo’s study in 2020 showed that sports drinks (pH 3.8) and apple flavoured thickened drinks (pH 3.5) had less of an effect on the disintegration time of tablets than roasted green tea (pH 6.5) and green tea flavoured thickened drinks (pH 6.6) [19]. Conversely in 2021 Matsuo compared the disintegration times of 4 medications (magnesium, senna, furosemide and aspirin) in 12 different solvents (including water, tea, juice and coffee) and concluded that different solvents did not differentially affect disintegration rates [17].

### The differential effect of thickened fluid on various medications

In 2020 Matsuo’s group investigated the disintegration time of 40 different tablets comparing non-immersed tablets to those immersed in 1% xanthan gum-based thickener, 3% xanthan gum-based thickener and a commercially available thickened drink for 1 min. They found that the medication being tested did differentially impact the effect of thickener on the disintegration rate [15]. It was not clear whether a particular property of some tablets contributed to this. Brave-Jose’s observational study in 2022 bore out similar results. They investigated 45 medicinal products and used a starch-based thickener. They found that some drugs were incompatible with thickener and observed the formation of clumps that did not dissolve. Specifically, they noted this for almagate, ibuprofen and macrogol [20].

Sugiura et al. [21] compared the disintegration time of control tablets with tablets that had been immersed in 0.9% w/v xanthan gum thickener for 1 min. Disintegration time did significantly increase for all 7 tablets when immersed in thickener. There was only a weak correlation between the duration of tablets control disintegration and duration after immersed in thickener though (*r* = 0.431).

### The effect of thickener on the therapeutic efficacy of some medications

In the first study of its kind, in 1984, Hupponen et al. conducted a double-blind study whereby volunteers received either digoxin or penicillin medication taken with either guar gum or a placebo preparation (wheat germ) mixed with water [22]. Serum and urine concentrations of digoxin were measured by radioimmunoassay. Serum penicillin concentrations were measured microbiologically according to Reeves et al. They found that the mean peak digoxin concentration was lower when the medication was taken with guar gum compared to placebo granules (0.05 < *p* < 0.1) but similar amounts of digoxin were recovered by 24 h in urine samples. A significantly lower mean peak penicillin concentration (*p* < 0.01) was obtained on guar gum compared to placebo. There was no difference in time to reach peak penicillin concentration. 24 h urine samples were not available for penicillin. It should be noted, however, that in this study guar gum was not being considered for its properties as a thickening agent (moreover as a treatment for diabetes) and thus it is not clear if the viscosity of the guar gum is similar to what is used in clinical practice for thickening. Furthermore, this was a small study (*n* = 10) only looking at two medications.

In 2015 Tomita et al. conducted an observational study on patients who were admitted to hospital wards during a 14 day period—who were all taking magnesium oxide (*n* = 147). They found that 21 patients were observed to be using thickening agents. Whether they disintegrated magnesium oxide into a small amount of water prior to adding thickener was observed, and the dose taken was observed. Whether they took additional laxatives was also observed. The mean dose of magnesium was 1705 mg/day in those who took a thickened fluid without disintegrating it first, 1122 mg/day in those who disintegrated into water and then added thickener. The average dose in those who took it with thin fluids was 1380 mg/day. 25% of patients who did not use thickener took an additional laxative. 33% who took magnesium with thickened fluids without disintegration, and 60% of those who took magnesium with thickened fluids after disintegration, used another laxative [23]. This study did not qualify whether thickener significantly affected the amount of magnesium used, or whether there was a statistically significant difference in the requirement for a second laxative. It is also not clear whether other clinical conditions that may have affected laxative use were accounted for.

In 2017 Tomita et al. performed a single-centre prospective cross-over study they compared the effect of immersion in xanthan gum Vs control on the therapeutic effect of mitglinide tablets (*N* = 5). They found a significant difference in blood glucose levels from administration to 90 min. The greatest difference in blood glucose levels was at 60 min (123 mg/dL for immersed tablets Vs 82 g/dL for non-immersed tablets. *p* < 0.01) [1]. This study corroborated findings from one the group did in 2016 assessing whether immersion in thickener impacted the therapeutic efficacy of another diabetic medication (voglibose) which once again demonstrated a significant difference in blood glucose levels between control and test group (immersed in 3% thickener for 10 min, *N* = 9) [24].

Interestingly in 2019, Tomita’s group demonstrated, like in vitro, the in vivo effects of immersion in thickener may differentially affect the therapeutic efficacy of different medications. In a single centre cross-over design study, they compared the time taken to reach a maximum systemic concentration of levofloxacin after emersion of tablets in thickener, jelly water and control. They found no significant difference in time taken to maximum system concentration and no significant difference in maximum systemic concentration [2].

## Discussion

There is an expanding field of in-vitro and in-vivo studies evaluating the potential therapeutic impact of thickeners on the medications that they are taken with. Variables such as the type of thickener (starch based, xanthan gum based or guar gum based), the viscosity of the thickened fluid and the duration tablets are immersed in thickener seem relevant. Several in-vitro studies have demonstrated that these variables significantly affect outcome measures including disintegration and dissolution rates. Whether these variables would significantly impact therapeutic outcomes in-vivo remains to be seen, although two out of three single-centre studies have demonstrated that the general concept of immersion in thickener does affect therapeutic outcomes [16, 24]. It should be noted however that all participants in these studies were healthy and so what the therapeutic impact on glycaemic control in a diabetic patient taking either mitglinide or voglibose with thickener in the long term remains to be seen.

The type of liquid used with thickener has demonstrated differing significance in various studies in vitro. Given the variation of outcomes in these experiments, and the potential clinical relevance (noting changing pH through the digestive tract) further investigation in this field may prove relevant. It may aid understanding of both the significance of the type of liquid that thickener is added to and the relationship between in-vitro and in-vivo experiments—where the pH of the liquid used is recorded and is comparable to that of the digestive tract. It may also be important to consider whether enzymes within the digestive tract are significant in terms of whether thickener effects therapeutic outcomes.

Both in-vitro and in-vivo studies seem to show that medications (and their therapeutic effect) may be differently affected by the use of thickener. Two in-vitro studies each reviewing 40 + medications have shown that immersion in thickener differentially effects disintegration rates and that several medicines form clumps that do not dissolve when immersed in thickener [19, 20]. The differential effect of medication choice on whether therapeutic impact is likely to be affected by thickener in vivo is also borne out by comparing the studies of Tomita et al. [16, 24, 25]. They demonstrated in two small single-centre studies that the therapeutic effect of two diabetes medications is impacted by immersion of tablets in thickener, whereas the systemic concentration of the antibiotic levofloxacin (and thus assumedly its therapeutic impact) is not affected.

Analogous to the studies reviewed here Wright et al. investigated the effect of a novel gel-based swallowing aid on systemic salicylate levels and platelet function when delivered with 300 mg aspirin tablets in a cross-over study of twelve healthy volunteers. This gel formation had been developed to ease the administration of tablets to patients with dysphagia. They found that when aspirin tablets were administered with the gel formation systemic salicylate levels were reduced and platelet function was increased. They concluded that bioequivalence could not be assumed [28]. Although not the focus of this review, the shared properties between this gel based swallowing aid and thickener should be considered (e.g. viscosity). On the other hand, although not the focus of this study, it is worth noting that two animal studies have not demonstrated a significant effect of the administration of medications with thickener on their bioavailability. Ilgaz et al. [29] found that in rabbits regardless of thickness level, administration of levetiracetam with thickening agents does not affect the bioavailability of the drug. Gotoh et al. [30] compared the administration of famotidine without food, with yoghurt and with thickener in rats. They found no difference in plasma concentrations in all 3 groups (with levels being taken up to 6 h after administration).

A limitation of this review is that the initial search used only two databases. Search terms included: dysphagia AND bioavailability OR absorption of medicines OR pharmacokinetics AND (with either) Parkinson’s/antibiotic/epilepsy/antiviral/thickened diet (EMBASE only). This was with a view to targeting our review of medicines whereby bioavailability is critical to efficacy. During the initial screening of titles/abstracts it became apparent that research into the effect of thickener on the bioavailability of oral medications is a small field and as such we did not exclude any studies based on oral medications being tested.

To conclude, these initial small single-centre studies suggest that thickener may affect the therapeutic efficacy of at least some medications. The overall clinical relevance of this remains to be seen but may be of particular significance when considering medications such as warfarin, digoxin and some anti-epileptic medications and antibiotics (with narrow therapeutic drug levels). Further larger-scale studies are required to evaluate what the therapeutic impact of thickener is on a much bigger range of medications, factoring in other variables that have been shown to be relevant in vitro including the type of thickener, the viscosity of thickener and duration of immersion. In the meantime, this review highlights the importance of doctors and speech and language therapists to consider carefully the necessity of oral thickener and the route of prescribed medications.

### Supplementary Information

Below is the link to the electronic supplementary material.

Supplementary file1 (DOCX 62 KB)
